# Mpox Airborne Transmission: A Systematic Review

**DOI:** 10.1002/rmv.70196

**Published:** 2026-08-10

**Authors:** Lucas Ferreira Theotonio dos Santos, Leonardo Javier Arcuri, Viviane Aparecida R. Sant’Anna, Patrícia Campos Couto, Guilherme de Paula P. Schettino, Luiz Vicente Rizzo, Henrique Andrade R. Fonseca

**Affiliations:** ^1^ Academic Research Organization Instituto Israelita de Ensino e Pesquisa Hospital Israelita Albert Einstein São Paulo São Paulo Brazil; ^2^ Secretaria de Ciência, Tecnologia, Inovação e Complexo da Saúde Ministério da Saúde Brasília Distrito Federal Brazil; ^3^ Escritório de Responsabilidade Social Hospital Israelita Albert Einstein São Paulo São Paulo Brazil

**Keywords:** aerosol, droplets, mpox, transmission, virus

## Abstract

Since May 2022, mpox has been reported across multiple nonendemic regions, with concurrent outbreaks in diverse geographic settings, raising concerns about broader international spread. However, evidence regarding the risk of airborne transmission remains limited. We conducted a systematic review to evaluate the risk of airborne transmission and the presence of viable viruses in air samples. PubMed, Web of Science, and the Cochrane Library were searched for studies published in English, including environmental studies, case reports, cross‐sectional studies, and cohort studies. Data extraction included study characteristics (authors, year, design, and setting), sample size, diagnostic methods, participant characteristics, follow‐up, type of respiratory particles assessed (droplets and/or aerosols), and key findings. Of 286 records identified, four studies met the inclusion criteria. All were environmental studies published between 2022 and 2023. MPXV DNA was detected in surface and air samples, with cycle threshold (Ct) values ranging from < 30 to < 45. However, none of the studies demonstrated consistent viral viability in aerosols or droplets sufficient to support airborne transmission. Overall, the available evidence is limited and does not support a clear risk of airborne infection. Further well‐designed studies in human settings are needed to clarify the potential for mpox transmission through respiratory routes.

**Systematic Review Registration:**

PROSPERO: CRD42023391123.

## 1 Introduction

Mpox, formerly known as monkeypox, is a zoonotic infection caused by an Orthopoxvirus. The virus was first identified in 1958 following outbreaks of a pox‐like illness in laboratory monkey colonies [1]. The first confirmed human case was reported in 1970 in the Democratic Republic of Congo, during intensified smallpox eradication efforts. Historically, transmission has been largely confined to endemic regions of Central and West Africa, with only sporadic cases reported elsewhere.

Since May 2022, however, mpox has been reported across multiple nonendemic regions, with concurrent outbreaks in diverse geographic settings [2], raising concerns about broader international spread [3, 4, 5]. Clinical presentations during these outbreaks have often been atypical, underscoring the importance of considering mpox in patients with compatible epidemiologic exposures, even when classic features are absent. In response to a rapid increase in infections caused by clade I monkeypox virus in Central Africa, the World Health Organization declared a second Public Health Emergency of International Concern on August 14, 2024.

The diagnosis is confirmed by polymerase chain reaction (PCR) because of its high sensitivity and specificity. Person‐to‐person transmission occurs primarily through direct contact with lesions and via respiratory droplets. The potential for airborne transmission remains uncertain; although evidence from animal experiments, observational human studies, and environmental sampling has been reported, a clear consensus has not been established [6, 7, 8, 9, 10].

Environmental studies have suggested the possibility of airborne transmission, often using viral viability as a surrogate; however, systematic epidemiologic investigations are needed to clarify this issue [9]. Airborne transmission is traditionally defined as the inhalation of aerosols containing particles smaller than 5 μm, typically occurring at distances greater than 1–2 m from an infected individual [11].

Respiratory particles, including droplets and aerosols, are generated during tidal breathing, coughing, and sneezing, producing particles of varying sizes that are expelled into the air [12]. Respiratory symptoms may facilitate the generation of virus‐containing aerosols, thereby increasing the potential for airborne transmission. However, the prevalence of respiratory manifestations in mpox remains uncertain, with published evidence ranging from isolated cases to larger case series reporting respiratory involvement [13].

A clearer understanding of the potential for airborne transmission of mpox will help inform public health policies and preventive strategies. In this context, this systematic review aimed to identify and synthesise available evidence on respiratory droplets and aerosols, and to assess the transmissibility of mpox through these routes.

## 2 Methods

This systematic review was conducted in accordance with the Preferred Reporting Items for Systematic Reviews and Meta‐Analyses (PRISMA) 2020 Statement [14]. The PRISMA 2020 checklist was consulted. The review protocol was registered in the International Prospective Register of Systematic Reviews (registration number: CRD42023391123).

### 2.1 Eligibility Criteria

#### 2.1.1 Types of Studies

We included case reports, cross‐sectional studies, case–control studies, cohort studies, and environmental studies.

#### 2.1.2 Population

Index cases were defined as adults (≥ 18 years), of any sex, from any country, with mpox confirmed by PCR laboratory methods.

#### 2.1.3 Outcomes

The primary outcomes were (1) viral viability in air samples assessed in the laboratory (e.g., viral culture and/or quantitative reverse‐transcription PCR) and (2) evidence of infection attributable to airborne transmission, defined as cases occurring in the absence of documented direct or indirect contact between the index case and the exposed individual.

### 2.2 Search Strategy

Two authors (H.A.R.F. and L.F.T.S.) conducted a pilot search to identify appropriate keywords and select relevant electronic databases. The search strategy was iteratively refined, as needed, by a third author (L.J.A.). The final search strategies for each database are provided in the Supporting Information S1.

We searched Web of Science, PubMed, and the Cochrane Library for studies published from the first reported human cases of mpox through February 2025. Search terms and strategies were tailored to the specifications of each database to optimise retrieval performance, including recall, precision, and the number needed to read.

Grey literature was not included in order to minimise the risk of bias in the interpretation of the findings.

### 2.3 Study Screening and Selection

We included published or accepted peer‐reviewed studies reporting MPXV detection in air samples. The primary composite outcome was defined as (1) viral viability in air samples assessed in the laboratory (e.g., viral culture in cells and/or quantitative reverse‐transcription PCR) and/or (2) infection attributable to airborne transmission, defined as occurring in the absence of documented direct or indirect contact between the index case and the exposed individual. Animal studies were excluded. Studies based on animal models for hypothesis generation were not considered eligible, given the uncertainty regarding the extent to which experimental conditions reproduce the generation and dispersion of aerosols and droplets associated with human mpox infection in real‐world indoor and outdoor environments.

**FIGURE 1:**
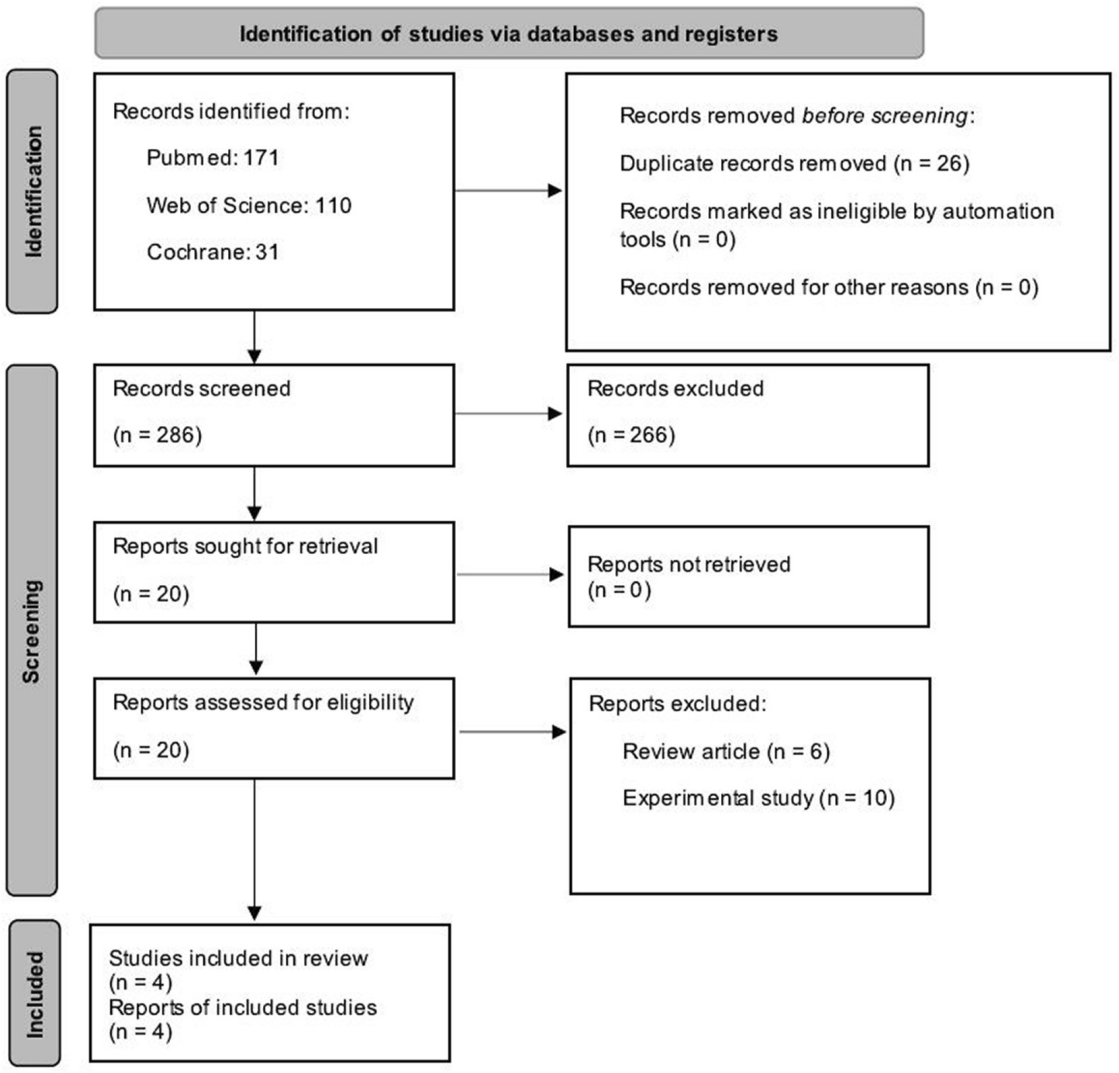
Study selection.

The study selection process was conducted independently by two authors (H.A.R.F. and L.F.T.S.), who screened the titles and abstracts of all identified records to determine eligibility for full‐text review. Full‐text assessment was performed without blinding to study authors, institutions, journals, or countries. Reasons for exclusion at the full‐text stage were systematically documented. Disagreements regarding study inclusion or exclusion were resolved through discussion between the two reviewers, with adjudication by a third reviewer (L.J.A.) when necessary. The number of studies included and excluded at each stage of the selection process is presented in a PRISMA flow diagram [14] flowchart model (Figure 1).

### 2.4 Data Extraction, Evaluation, and Synthesis

The two reviewers responsible for study selection were trained to ensure standardized data extraction. We used a structured data extraction form adapted from Cochrane and recorded the data in a Microsoft Excel spreadsheet. Extracted variables included study title, authorship, year of publication, study design and methods, participant characteristics, study setting, type of respiratory particles evaluated (droplets and/or aerosols), and key findings from the included studies.

### 2.5 Quality Assessment

To our knowledge, no validated risk‐of‐bias or quality assessment tool is specifically designed for the appraisal of environmental studies of the type included in this review. Accordingly, a formal quality assessment using a standardized instrument was not performed. Instead, we undertook a structured qualitative appraisal of each study, considering key methodological domains such as sampling methods, laboratory procedures, outcome measurement (e.g., viral detection and viability), and completeness of reporting.

## 3 Results

### 3.1 Study Selection

Between December 2024 and February 2025, we identified 286 records across the three prespecified databases (PubMed, 1975–2025; Web of Science, 1945–2025; Cochrane Library, 1950–2025). After screening titles and abstracts, 22 articles were selected for full‐text assessment. Following detailed evaluation, four studies met the eligibility criteria and were included in the final analysis (*n* = 4) [15, 16, 17, 18]. All included studies met the prespecified eligibility criteria and were classified as environmental studies (Figure 1). No case–control studies, cohort studies, or case reports providing evidence of confirmed airborne transmission were identified.

### 3.2 Risk of Bias

Selection bias may be present, as the definition of ‘proven airborne transmission’ relied on the interpretation of the original study investigators and the judgement of the review team. No additional major sources of bias were identified.

### 3.3 Study Characteristics

All four included studies were environmental investigations. As detailed in Table 1, these studies sampled multiple environmental surfaces, including air samples, and in some cases incorporated additional sampling sources. Table 1 summarises the key characteristics and principal findings of each study.

**TABLE 1:** Evaluation of detection of Mpox virus and risk of infection.

Article title	Doi	Principal author	Year	Biologic sample	Diagnosis method	Mpox DNA cycle threshold [ct] for positive PCR	Findings	Commentary
Air and surface sampling for monkeypox virus in a UK hospital: An observational study	10.1016/S2666‐5247(22)00257‐9.	Susan Gould	2022	Air and surface	Viral DNA detection by qPCR and culture in vero C1008 cells	< 30	MPXV DNA was detected in surfaces [ct range (24.7–37.4)] swab samples taken from patient bedrooms and bathrooms. The positive results included samples from areas not likely to have been directly touched by patients, such as the air vent above the door. Evidence of replication‐competent virus from two samples: From an air sample [ct range (17.5–35.5)] collected during a bedding change and from a swab sample of an anteroom floor.	None of the patients in the rooms sampled had respiratory symptoms during the in‐hospital stay, at least according to the original report. Given that this is the case, the transmissibility through air may be underrepresented and its assessment compromised, as the viral load in the respiratory tract may be lower in those patients without symptoms.
Monitoring monkeypox virus in saliva and air samples in Spain: a cross‐sectional study	10.1016/S2666‐5247(22)00291‐9	Bruno Hernaez	2022	Saliva, exhaled droplets, and aerosols	Viral DNA detection by qPCR and culture in BSC‐1 cells	< 35	In droplets exhaled by mpox patients and captured inside masks, monkeypox virus DNA was found in 71% of samples, with two of these samples containing the infectious virus. In aerosols collected, monkeypox virus DNA was present in 64% of samples [ct range (30–29)]. No infectious virus was recovered from those aerosols.	Respiratory symptoms just in some of their patients—5 out of 46 had cough, and 6 out of 46 had any respiratory symptoms. Nevertheless, no viable virus was retrieved from air samples for detection risk for infection.
Viable mpox virus in the environment of a patient room	10.1016/j.ijid.2023.03.016	Kalisvar Marimuthu	2023	Air, surface dust, and water sampling	Viral culture and PCR	No report	Viable MPXV was found in surface and dust samples, but no viable virus was detected in air or water samples. No data regarding ct measures was reported.	No respiratory symptoms were present, at least as reported. This may underrepresent viability in air samples for replication and infection risk.
Detection of mpox virus in ambient air in a sexual health clinic	10.1007/s00705‐023‐05837‐z.	Joren Raymenants	2023	Air, surface	qPCR	< 45	MPXV was detected in air samples of 3 out of 6 positive patients for mpox disease. Air samples for infected patients had a median of 33.14 for cycle threshold [ct, IQR (31.58–35.13)].	No test of viability was performed (such as culture) and it should be added to the analysis mainly because the ct of qPCR was high denoting low viral load in the sample, so the evaluation of viral viability would be of most importance.

### 3.4 Outcomes Measures

The authors evaluated whether air sampling could detect MPXV across a range of cycle threshold (Ct) values using real‐time quantitative polymerase chain reaction (qPCR), with Ct values often considered a surrogate marker for viral viability. Reported Ct cutoffs vary across studies, although values < 40 to < 45 are generally accepted as indicative of a positive result [15, 19]. However, the four studies included in this review applied Ct thresholds ranging from < 30 to < 45, limiting the comparability of MPXV detection across studies. Notably, MPXV was detected in both surface and air samples collected from hospital rooms previously occupied by patients with mpox, with Ct values ranging from 17 to 37.4. Viral viability was further assessed using cell culture techniques [16, 17, 18]. (Table 1). Only one study included Mpox patients with respiratory symptoms (13%), with no impact on the results concerning MPXV viability [17].

### 3.5 Ethics

No personal data were collected. Ethics approval was not required for this systematic review.

## 4 Discussion

Our findings indicate that the available literature provides limited evidence regarding the risk of MPXV transmission through respiratory droplets and aerosols across different settings. Despite a comprehensive search of three databases, only four studies met the inclusion criteria by assessing MPXV in air or droplet samples, with quantification by Ct values and/or viral culture. Overall, these studies yielded heterogeneous and inconclusive results regarding the potential for transmission via contaminated droplets or aerosols.

After mpox infected patients' assistance, surface across various areas, including occupied patient rooms, health‐care workers' personal protective equipment (PPE) after use, and the areas where PPE was analysed, revelling that most surface samples tested positive (93% of samples collected) for the presence of monkeypox DNA, indicating a significant level of contamination. In addition, a portion of the air samples (25%) collected showed evidence of the virus, with some of these taken before and during a bedding change in a patient's room (a source of aerosolisation) [18]. This suggests that certain activities, such as bedding changes, may increase the likelihood of airborne transmission. Furthermore, they successfully isolated replication‐competent viruses from a subset of the samples, underscoring the potential to remain infectious in the environment. However, no patient in this study presented respiratory symptoms. Hence, the possibility of the aerosols being generated by the patient is doubtful, and bedding change is probably the primary source of the aerosols in this circumstance.

In other hands, Raymenants et al. [15] observed that ambient air PCR tests yielded positive results for all six patients infected with MPXV, with each of the four tests confirming the presence of the virus. In contrast, among the patients who were not infected, three ambient air samples also tested positive; however, these samples exhibited higher Ct values, suggesting a lower viral load or potential contamination. Further analysis through genomic sequencing of samples from two infected patients revealed that the viral sequences found in the air closely matched those in the clinical samples taken from these patients. This finding underscores the potential for airborne transmission, although no viability test was performed for the virus identified in the air sample.

During the 2022 mpox outbreak (May–July), a study conducted at two health centres in Madrid identified high levels of MPXV DNA in the saliva of most patients, with viable virus detected in a substantial proportion (67%) of samples. A positive correlation was observed between symptom severity and salivary viral load. In addition, MPXV DNA was detected in respiratory droplets exhaled by patients (including samples collected from the inner surface of masks) and in aerosol samples obtained in clinical settings [17]. However, viable viruses were not recovered from the aerosol samples.

Marimuthu et al. [16] conducted an observational study to assess environmental contamination, including air, surfaces, dust, and water, in a room occupied by a patient with confirmed mpox across different stages of illness. The diagnosis was established by polymerase chain reaction testing of throat swabs and skin lesions. Environmental sampling was performed in a negative‐pressure isolation room equipped with high‐efficiency air filtration and subjected to routine surface disinfection. Samples were collected serially over the course of the illness, from early to later stages. The highest levels of contamination in air, surfaces, and dust were observed during the early phase, with a progressive decline over time. By day 21 of illness, contamination levels had markedly decreased. Notably, viable MPXV was successfully isolated from surface and dust samples, indicating the presence of infectious virus in these matrices. In contrast, no viable virus was detected in air samples.

A recently published Personal View [6] highlights the need for further studies to determine whether MPXV can be transmitted through airborne routes. A systematic review published in 2019 [20] asserted in its introduction that transmission occurs through respiratory droplets. However, the supporting evidence was limited, relying primarily on secondary sources, including review articles and a 1988 World Health Organization bulletin [21], which is not as definitive as stated in the text. In a retrospective study, Adler and colleagues [22] described seven cases of mpox diagnosed in the United Kingdom between 2018 and 2021. The series included a health care worker with presumed occupational acquisition and a patient who acquired infection abroad and subsequently transmitted it to two household contacts. MPXV DNA was detected in skin lesions in all patients, and three individuals demonstrated prolonged viral shedding from the upper respiratory tract, lasting at least 3 weeks. Despite this evidence of respiratory tract shedding, the available data remain insufficient to support airborne transmission as a plausible route of infection.

Airborne transmission should be considered within a multidisciplinary framework that integrates epidemiological observations with the physical principles governing aerosol generation, transport, deposition, and viral persistence [23, 24]. Experimental studies have demonstrated that MPXV remains viable in aerosols under controlled laboratory conditions, while aerosol dynamics modelling suggests that virus‐containing particles may remain suspended and disperse beyond close‐contact distances, particularly in poorly ventilated indoor environments and among individuals with high viral loads [6]. Although routine respiratory activities, including quiet breathing, can generate aerosols [25, 26] that may contain infectious viral particles, the epidemiological significance of this route for MPXV transmission remains uncertain. Although these findings do not establish airborne transmission as a predominant route in real‐world settings, they provide a coherent mechanistic basis supporting its biological plausibility and reinforce the need to integrate physical and epidemiological evidence when assessing MPXV transmission.

The epidemiological contribution of asymptomatic and presymptomatic MPXV infection remains uncertain. Evidence from other viral infections has shown that symptom‐based surveillance may underestimate transmission by failing to identify infected individuals without clinical manifestations [27]. Likewise, none of the studies included in this review evaluated asymptomatic individuals, which may have led to an underestimation of the potential contribution of aerosols and respiratory droplets to MPXV transmission. Although asymptomatic infection appears to be uncommon, the detection of MPXV in asymptomatic and presymptomatic individuals suggests that these infections may contribute to onward transmission [28, 29, 30], posing important challenges for case detection, contact tracing, and infection prevention strategies. Future studies integrating aerosol physics, environmental sampling, viral viability, and epidemiological investigations are needed to better define the contribution of airborne transmission to MPXV spread, including asymptomatic populations.

### 4.1 Study Limitations

Several limitations should be acknowledged. First, the available evidence is constrained by the paucity of environmental studies capable of detecting and confirming viable MPXV in aerosol and droplet samples, with most investigations relying on surrogate markers such as PCR‐based detection rather than viral culture. Second, there is a notable absence of well‐designed epidemiologic studies providing robust, causal evidence linking respiratory exposure to subsequent transmission. Third, none of the included studies evaluated asymptomatic individuals. Given that MPXV has been detected in asymptomatic and presymptomatic infections, their exclusion may have introduced bias by underestimating the potential contribution of aerosols and respiratory droplets to transmission. Together, these limitations substantially restrict the strength of inference regarding the contribution of airborne and droplet‐mediated transmission to MPXV spread.

### 4.2 Study Implications

Further high‐quality studies are needed to clarify the risk of mpox transmission through airborne routes, including droplets and aerosols generated by infected individuals. In the absence of definitive evidence, a precautionary approach is warranted. This includes appropriate clinical management of patients with active mpox, implementation of risk‐reduction strategies to protect health care workers from potential exposure to respiratory particles, and optimization of health care environments to mitigate the risk of transmission.

In conclusion, available evidence remains insufficient to establish the epidemiological importance of airborne transmission in mpox. However, emerging experimental data and aerosol dynamics support its biological plausibility under specific conditions. Well‐designed multidisciplinary studies integrating epidemiological, environmental, virological, and physical evidence are needed to clarify the contribution of this transmission route and to guide future public health recommendations.

## Author Contributions

H.A.R.F. designed the study and wrote the manuscript. L.F.T.S, L.J.A, and V.A.R.S were responsible for the data synthesis strategy and manuscript writing. P.C.C, G.P.S, and L.V.R provided critical insights and wrote the manuscript. All authors approved the final version of the manuscript.

## Funding

The study was designed and led by an academic steering committee and sponsored by a grant from the Brazilian Ministry of Health and Hospital Israelita Albert Einstein as part of the NETPOX Programme for Mpox evidence—PROADI‐SUS (grant number, NUP‐ 25000.165135/2022‐64).

## Conflicts of Interest

The authors declare no conflicts of interest.

## Supporting information


Supporting Information S1

## Data Availability

The data that support the findings of this study are available from the corresponding author upon reasonable request.
