# Supplementary material for: Mpox Airborne Transmission: A Systematic Review
Source: Rev Med Virol. 2026 Aug 10;36(5):e70196. doi: 10.1002/rmv.70196 (PMC13457963; doi:10.1002/rmv.70196)
Supplement: Supplementary file 1 [file RMV-36-e70196-s001.docx]

SUPPLEMENTARY MATERIAL

MPox Systematic Review Strategy

Web of Science

| Search | Query |
| --- | --- |
| #1. | “monkeypox” or “monkey pox” or “mpox” (Topic) |
| #2. | “aerosol” or “particle” or “ambient” or “air” (Topic) |
| #3. | #1 and #2 |

Pubmed

| Search | Query |
| --- | --- |
| #1. | (("Monkeypox virus"[Mesh]) OR ("Mpox (monkeypox)"[Mesh]) OR ("Monkey pox")) |
| #2. | ("carrier"[Title/Abstract] OR "virion"[Title/Abstract] OR "virions"[Title/Abstract] OR "droplet*"[Title/Abstract] OR "nuclei"[Title/Abstract] OR "air"[Title/Abstract] OR "aerosol*"[Title/Abstract] OR "liquid*"[Title/Abstract] OR "fluid*"[Title/Abstract] OR "saliva*"[Title/Abstract] OR "bioaerosol"[Title/Abstract] OR "bio-aerosol"[Title/Abstract] OR "aeroallergen"[Title/Abstract] OR "aero-allergen"[Title/Abstract] OR "allergen*"[Title/Abstract] OR "pollution*"[Title/Abstract] OR "particulate"[Title/Abstract] OR "particulates"[Title/Abstract] OR "particle"[Title/Abstract] OR "particles"[Title/Abstract] OR "nanoparticle"[Title/Abstract] OR "nanoparticles"[Title/Abstract] OR "nano-particle"[Title/Abstract] OR "nano-particles"[Title/Abstract] OR "ultrafine"[Title/Abstract] OR "ultra-fine"[Title/Abstract] OR "ultra-fines"[Title/Abstract] OR "microscopic"[Title/Abstract] OR "particulate matter"[Title/Abstract] OR "PM"[Title/Abstract] OR "PM1"[Title/Abstract] OR "PM2.5"[Title/Abstract] OR "PM10"[Title/Abstract] OR "TSP"[Title/Abstract] OR "UFP"[Title/Abstract] OR "smoke"[Title/Abstract] OR "dust"[Title/Abstract] OR "dusts"[Title/Abstract] OR "pollen"[Title/Abstract] OR "pollens"[Title/Abstract] OR "spore"[Title/Abstract] OR "spores"[Title/Abstract] OR "mist"[Title/Abstract] OR "mists"[Title/Abstract] OR "vapour"[Title/Abstract] OR "vapours"[Title/Abstract] OR "vapor"[Title/Abstract] OR "vapors"[Title/Abstract] OR "haze"[Title/Abstract] OR "smog"[Title/Abstract] OR "contamina*"[Title/Abstract]) |
| #3. | ("environment*"[Title/Abstract] OR "airborne"[Title/Abstract] OR "air-borne"[Title/Abstract] OR "atmospher*"[Title/Abstract] OR "aerodynamic"[Title/Abstract] OR "aero-dynamic"[Title/Abstract] OR "transmit*"[Title/Abstract] OR "emit*"[Title/Abstract] OR "emission*"[Title/Abstract] OR "pathway"[Title/Abstract] OR "path"[Title/Abstract] OR "route"[Title/Abstract] OR "distanc*"[Title/Abstract] OR "presist*"[Title/Abstract] OR "expos*"[Title/Abstract] OR "carry"[Title/Abstract] OR "spread*"[Title/Abstract] OR "spray*"[Title/Abstract] OR "stream*"[Title/Abstract] OR "jet"[Title/Abstract] OR "plume"[Title/Abstract] OR "flow*"[Title/Abstract] OR "airflow"[Title/Abstract] OR "current"[Title/Abstract] OR "settl*"[Title/Abstract] OR "suspen*"[Title/Abstract] OR "resuspen*"[Title/Abstract] OR "propagat*"[Title/Abstract] OR "penetrat*"[Title/Abstract] OR "accumulat*"[Title/Abstract] OR "concentrat*"[Title/Abstract] OR "dilut*"[Title/Abstract] OR "dispers*"[Title/Abstract] OR "diffus*"[Title/Abstract] OR "drift*"[Title/Abstract] OR "float*"[Title/Abstract] OR "scatter*"[Title/Abstract] OR "project*"[Title/Abstract] OR "trajector*"[Title/Abstract] OR "shed*"[Title/Abstract] OR "travel*"[Title/Abstract] OR "transport*"[Title/Abstract] OR "infect"[Title/Abstract] OR "infected"[Title/Abstract] OR "infecti*"[Title/Abstract] OR "sneez*"[Title/Abstract] OR "cough*"[Title/Abstract] OR "breath*"[Title/Abstract] OR "talk*"[Title/Abstract] OR "speak*"[Title/Abstract] OR "speech*"[Title/Abstract] OR "sing*"[Title/Abstract] OR "blow"[Title/Abstract] OR "respira*"[Title/Abstract] OR "expira*"[Title/Abstract] OR "inhal*"[Title/Abstract] OR "exhal*"[Title/Abstract] OR "dry"[Title/Abstract] OR "dryer*"[Title/Abstract] OR "evaporat*"[Title/Abstract] OR "flush*"[Title/Abstract] OR "splash*"[Title/Abstract] OR "ventilat*"[Title/Abstract]) |
| #4. | #1 AND #2 AND #3 |

Cochrane

| Search | Query |
| --- | --- |
| #1. | “monkeypox” or “monkey pox” or “mpox” [Title Abstract Keyword] |
| #2. | “virion” or “virions” or “virus” or “droplet” or “aerosol” or “particle” or “ambient” or “air” [Title Abstract Keyword] |
| #3. | #1 and #2 |
